# Medication safety in acute care in Australia: where are we now? Part 1: a review of the extent and causes of medication problems 2002–2008

**DOI:** 10.1186/1743-8462-6-18

**Published:** 2009-08-11

**Authors:** Elizabeth E Roughead, Susan J Semple

**Affiliations:** 1 Quality Use of Medicines and Pharmacy Research Centre, Sansom Institute, University of South Australia, GPO Box 2471, Adelaide, 5001

## Abstract

**Background:**

This paper presents Part 1 of a two-part literature review examining medication safety in the Australian acute care setting. This review was undertaken for the Australian Commission on Safety and Quality in Health Care to update a previous national report on medication safety conducted in 2002. This first part of the review examines the extent and causes of medication incidents and adverse drug events in acute care.

**Methods:**

A literature search was conducted to identify Australian studies, published from 2002 to 2008, on the extent and causes of medication incidents and adverse drug events in acute care.

**Results:**

Studies published since 2002 continue to suggest approximately 2%–3% of Australian hospital admissions are medication-related. Results of incident reporting from hospitals show that incidents associated with medication remain the second most common type of incident after falls. Omission or overdose of medication is the most frequent type of medication incident reported. Studies conducted on prescribing of renally excreted medications suggest that there are high rates of prescribing errors in patients requiring monitoring and medication dose adjustment. Research published since 2002 provides a much stronger Australian research base about the factors contributing to medication errors. Team, task, environmental, individual and patient factors have all been found to contribute to error.

**Conclusion:**

Medication-related hospital admissions remain a significant problem in the Australian healthcare system. It can be estimated that 190,000 medication-related hospital admissions occur per year in Australia, with estimated costs of $660 million. Medication incidents remain the second most common type of incident reported in Australian hospitals. A number of different systems factors contribute to the occurrence of medication errors in the Australian setting.

## Background

Use of medications is central to modern health care, and nearly all patients visiting a hospital will receive one or more medicines during their hospital stay or upon discharge. While in the majority of cases medicines use will result in the desired outcome, medicines are not without risk, and problems or unexpected outcomes may arise.

As medicines are taken so commonly, sometimes problems can occur in their prescription, dispensing and administration which can be termed "*medication incidents*". A proportion of these medication incidents result in patient harm and are called "*adverse drug events*" (ADEs). Some ADEs result from the manner in which the medication is used (such as an error or system failure). Other ADEs are termed "*adverse drug reactions*" and can result from the pharmacological properties of the medication itself when it is taken alone or in combination with other medications. Adverse events associated with medications are common, affect a substantial number of people and contribute a significant burden to health care costs.

In 2000, the Australian Council for Safety and Quality in Health Care was established by the Australian Health Ministers to provide a focus of national leadership in tackling the issues of patient safety. As part of its work, in 2002 the Council commissioned a literature review of medication safety in the *Second National Report on Patient Safety Report – Improving Medication Safety *[1]. Amongst the findings of the review was that 2–3% of all hospital admissions in Australia were medication-related. A range of errors and system failures including errors in prescribing, administration and dispensing were found to occur in hospitals in Australia. There was limited Australian research on the causes of these errors although it was recognised that most errors resulted from a series of system failures rather than the actions of particular individuals. Some commonly associated factors identified included a lack of robust systems for prescription or ordering of medications and problems in the transfer of patient information between hospital and community settings. Evidence was found to support the use of a range of strategies to improve medication safety including computerised (electronic) prescribing with decision support, adverse drug event alerting systems, bar coding, clinical pharmacist services, services to improve information transfer between different settings and individual patient medication supply in hospitals. Careful implementation of computerised prescribing with clinical decision support systems in Australia was identified as a priority. However, it was recognised that there was an urgent need for more research examining the implementation and effectiveness of the various strategies in the Australian setting.

In 2006, the former Council was replaced by the Australian Commission on Safety and Quality in Health Care. The Commission's roles include the leadership and coordination of strategies to improve safety and quality in health care through identifying issues and policy directions, providing recommendations for action and advice to Health Ministers and publicly disseminating information on safety and quality [2].

The current Commission required an updated review to examine current trends in medication safety problems and progress in research on contributing factors to these problems. There was also a need to review more recent developments in research examining the implementation of, and evidence base for, strategies to improve medication safety in the Australian setting.

This paper presents Part 1 of this two-part review. Part 1 examines the extent and causes of medication incidents and adverse drug events in acute care in Australia to 2008. It is hoped this information will inform policy makers, health care professionals, managers and researchers about the areas in which significant problems with medication safety continue.

Part 2 examines the evidence for practices to improve safety in the Australian setting, barriers and facilitators to the implementation of these strategies and priorities for further research and policy-development. Part 2 is presented as a separate paper.

## Methods

### Search strategy

A literature search was undertaken to identify studies conducted in the acute health care setting in Australia since the time of publication of the former Australian Council for Safety and Quality in Health Care *Second National Report on Patient Safety – Improving Medication Safety *[1]. Searches were primarily undertaken by the New South Wales (NSW) Medicines Information Centre St Vincent's Hospital, Darlinghurst, NSW.

The search strategy for Part 1 of the review was designed to identify studies undertaken in Australia from 2002 to 2008 on the extent and causes of medication incidents and adverse drug events in acute care.

Searches were conducted in March and April 2008 in Medline (1950 – March Week 1 2008), EMBASE (1980 – March Week 1 2008), Pre-Medline and CINAHL (1982 – April Week 2 2008) using criteria relevant to the general headings in the former Council's *Second National Report on Patient Safety – Improving Medication Safety *[1]. All searches were limited to 2002–2008.

Search terms used included adverse drug event, adverse drug reaction, Australia, Australian, drug, error, event, exp adverse drug reaction reporting systems, exp Australia, exp drug surveillance program, exp drug therapy, exp hospital, exp hospitalization, exp hospitals, exp medication error, exp medication systems, exp patient safety, exp physician's practice patterns, exp quality assurance, exp safety management, healthcare, hospital, incident, medication errors, medication:, medication?, medicine:, medicine?, misadventure, mishap, mistake, problem. The exp (explode) function was used in the relevant databases to search for the subject heading as well as any more specific terms related to that subject heading. This expanded the results to include records about the broader topic and related topics.

The database search was supplemented with review of relevant reports and resources on the Australian Commission on Safety and Quality in Health Care website  including publications of the former Australian Council for Safety and Quality in Health Care and incident reports from State Government sites.

### Selection of studies for review

This review focussed on the acute care setting in Australia, studies undertaken in the community setting were excluded. Studies included in examining the extent and causes of medication incidents and adverse drug events at the systems level were:

- adverse drug event monitoring studies;

- medication incident monitoring studies (including studies where medication incidents were reported on as a subset);

- quantitative reports of medication incidents (including prescription errors, dispensing errors, administration errors);

- qualitative studies that examined causes of medication incidents (prescribing, administration and medication management deficiencies).

Where appropriate summary data tables from the former Council's *Second National Report on Patient Safety – Improving Medication Safety *[1] were updated with information from new studies and included in the review.

Case reports of medication errors leading to near misses or adverse drug events were excluded, as were adverse events or incidents specific to only one type of medicine.

## Results and Discussion

### The extent of medication-related hospital admissions

Medication-related hospital admissions represent problems with medications which may originate either within the community or within a hospital. Previous studies had indicated between 2% and 3% of all admissions were medication-related. Two new studies, published since 2002 give additional insight into the incidence of medicine-related hospital admissions in Australia [3,4]. One used the hospital morbidity records to determine the incidence of adverse drug reactions, finding 1.3% of admissions were associated with an adverse drug reaction at the time of the admission and that required treatment [3]. Another 0.3% of admissions had an adverse drug reaction identified at the time of admission, but not treated. A further 1.2% of admissions were associated with an adverse drug reaction that occurred during hospital stay [3]. Use of morbidity records alone is likely to under-estimate the incidence of these events as it has been demonstrated that while accurate, the adverse drug reaction codes are under-reported [5]. The second study assessed the incidence of adverse drug reactions in oncology patients [4]. It included both adverse drug reactions present on admission and occurring during hospital stay, finding that 74% of oncology admissions were associated with an adverse drug reaction, with a median of 2 adverse drug reactions per admission. Overall 47% were potentially preventable. Patients were asked to rate the impact of the adverse drug reaction on a scale from 0 (no impact at all) to 6 (totally changed my life). Fifty three percent of patients rated the reaction at four or above with 19% rating the adverse drug reaction as "totally changed my life" [4].

The inclusion of these studies with the results from the previous *Second National Report on Patient Safety Improving Medication Safety *[1] (See table S1 – Additional file 1) still suggests an overall rate of medicine related hospital admissions in Australia of between 2% and 3%.

Attendances to the emergency department have also been included (See table S1 – Additional file 1). Since 2002, there has been one new study undertaken in the paediatric population [6] and one study in the adult population [7]. Results from the general population of 8.3% of adult emergency attendances (not admitted) being medicine related [8] pertain to data collected in 1993. A more recent study found an adverse drug reaction rate of 1.4% in emergency department attendances (including those subsequently admitted) and another 18 adverse drug events documented [7], but an overall incidence rate of emergency department attendances due to medication related problems was not able to be calculated. The emergency department attendance rate of medicine-related attendances is not dissimilar to the community estimates that 10.4% of people attending a general practitioner had had an adverse drug event in the previous six months [9].

Preventability estimates for medication-related hospital admissions and adverse drug reactions associated with hospitalisation suggest between one third and three quarters are potentially preventable (Table 1).

**Table 1:** Preventability of adverse medicine events associated with hospitalisation or admissions due to medication-related problems

		**Total number of medicine-related problems or admissions**	**Percentage considered definitely avoidable**	**Percentage considered probably or possibly avoidable**	**Percentage considered probably not or definitely unavoidable**
Titchen et al., 2005 [35]	Hospital Paediatric NSAID ADRs	25	36%		

Easton et al., 2004 [36]	Paediatric admissions	81	46.9%		30.9%

Easton-Carter et al., 2003 [6]	Paediatric emergency department attendances	187	51.3%		36.9%

Chan et al., 2001 [37]	Geriatric admissions	73	53.4	23.3	23.3

Lau et al., 2004 [4]	Hospital Oncology ADRs	454	1.6%	46.1%	53.4%

Dartnell et al 1996 [38]	General admissions	55*	5%	60%	35%

Sarkawi et al, 1995 [39]	Medical admissions	35*	23%	46%	31%

Easton 1998 [40]	Paediatric admissions	48*^+^	#	67%	29%

Ng 1996 [41]	Geriatric admissions	31	3%	29%	68%

* – overdose excluded; # – category not used; + – 2 cases not assessable. ADRs = adverse drug reactions; NSAID = non-steroidal anti-inflammatory drug. Note: estimates of adverse drug event preventability in the community from one study were 23% [9].

Two other studies give insight into adverse drug reactions during hospitalization, but not incidence figures. These used the hospital morbidity coding records for Western Australia [10,11]. One found the trend over time in adverse drug reactions associated with hospital admissions had increased five-fold between 1981–2002, from 2.5 per 1000 person years to 12.9 per 1000 per years [10]. This is similar to what was reported from South Australia [1], with the South Australian results showing a strong correlation with medication use [12], suggesting the increase is related to changes in medication use rather than an increased incidence of events. The second study reported "repeat" adverse drug reactions, finding that "repeat" adverse drug reaction-related hospitalisations increased at a faster rate than the overall rate of adverse drug reaction hospitalisations, with estimates that repeat adverse reaction hospitalisations accounted for 30% of all adverse drug reaction hospitalisations by 2003 [11]. This result should be interpreted cautiously. "Repeat" adverse drug reactions included another admission for an adverse drug reaction not a repeat admission for the same adverse drug reaction. Further, the results have not been adjusted for length of follow-up. Cytotoxics and hormones accounted for a larger proportion of repeat admissions than first admissions [11], which may indicate that treatment patterns for the underlying diseases impacted on the overall population available for repeat admissions. High rates of adverse drug reactions in the oncology population have been reported [4].

Overall, these data suggest medication-related hospital admissions still represent a significant burden on the Australian community. Based on annual hospital admissions data for 2006–07 in which there were 7.6 million separations, it can be estimated that there are approximately 190,000 medicine related hospital admissions in Australia each year with an estimated cost of $660 million.

### Adverse events associated with intra-hospital transfers

Evidence also highlights the potential problem of medication errors occurring as a result of intra-hospital transfer, particularly after hours. A 2006 study assessing adverse events occurring within 72 hours of discharge from the intensive care unit found 17 (10%) of 167 discharges were associated with an adverse event, with 52% preventable. While not focused specifically on medications, 47% of the adverse events were related to fluid management. Eighty-two percent of the discharges associated with adverse events were discharges that occurred after hours or at weekends [13].

### Medication incidents in acute care

Incident reporting from Western Australia and New South Wales has been compared with that from South Australia reported in the *Second National Report on Patient Safety-Improving Medication Safety *(Table 2). Medication incidents remain the second most frequent incident reported, with falls being the predominant incident. As a proportion of all incidents, medication incidents were similar across WA and SA, with a lower percentage reported in NSW. Omission and overdose remain the most common type of medication incident, with failure to read or misreading the chart and failure to follow protocol the most commonly cited causes. The majority of medication incidents cause no harm or only minor harm. Analgesics and anticoagulants appear to be the medicines most commonly implicated. The peak time of day for medication incidents is at 0800 – 0900 hours and 2000 – 2100 hours in both WA and NSW. Nurses reported the majority of incidents.

**Table 2:** Medication incident reports, SA, WA and NSW

	**SA (pre 2002) **[1]	**WA 03/04 **[42]	**WA 04/05 **[43]	**WA 05/06 **[44]	**NSW 05/06 **[45]
**Number of incidents**	26999	23189	21693	20799	123404

**Medication incidents**	7155 (26.5%)	23.5%^#^	24.0%^#^	5068 (24.4%)	17367 (14.1%)

**Outcome**					

No injury	69%^@^	87.0%	85.0%	85.0%	82%*

**Most common type of medication incident**					

Omission	27.9%	36.0%	36.0%	37.0%	

Overdose	19.5%	18.0%	17.0%	19.0%	

Prescription or order error				14.0%	

Unclear or incomplete order				6.0%	

Dispensing error	3.3%			2.0%	

**Most common reason cited for medication incident**					

Failure to read or misread	52%	49.0%		36.0%	

Failure to follow policy		23.0%		26.0%	

**Medicines implicated**					

	Cardiovascular; Analgesics, CNS, Endocrine, Antibiotics	Analgesics; Anticoagulants Diuretics; Respiratory; Proton Pump inhibitors		Analgesics; Anticoagulants; Diuretics; Steroids	Analgesics; Anticoagulants; Insulins; Diuretics

@ = none or minor; # = estimated from graph; * = Severity Assessment Code (SAC) 3 or SAC 4

A South Australian survey of 186 doctors and 587 nurses (70.7% and 73.6% response rate respectively) found that 100% of nurses stated they always reported a medicine error that required giving a patient corrective treatment, compared to only 40% of the doctors, while less than 20% of each group stated they reported near miss medication errors [14]. Lack of feedback, the form taking too long to complete, the perception that the incident was trivial and the ward being busy, were the most common reasons cited for not reporting an incident [14].

Three other published studies which give some insight into medication incident rates in specific areas of practice are summarised in Table 3. These studies were conducted in anaesthetics, intensive care and in a district hospital setting.

**Table 3:** Medication incident rates in specific practice areas

	**Type of incident**	**Denominator**	**Medication incidents (n)**	**Rate**
Freestone et al., 2006 [46]	Anaesthetic incidents	4441 procedures	10	0.2% of procedures

Chacko et al., 2007 [47]	Critical incidents in intensive care	8346 ICU days	42	0.5 per 100 ICU days

Parke 2006 [48]	Medication use in a district hospital	24174 medication dispensings	425	1.8%

### Prescribing errors in acute care

#### Incidence of prescribing errors

Since 2002, one new study has assessed the overall incidence of prescribing errors on discharge prescriptions, comparing hand written discharge medication prescriptions with computer generated discharge prescriptions, finding much higher rates of error with computerised systems (11.6%) compared with hand written systems (5%) (p < 0.001) (Table 4). Additional errors which appeared to be associated with computer systems were excessive duration (primarily associated with antibiotic durations extended because of the default quantity in the prescribing software), dosing errors and inclusion of medicines intended to be ceased [15].

**Table 4:** Types of errors: Prescription errors: Australian hospitals 1985–2007

**Reference**	**Number of prescriptions or charts audited**	**No. of errors detected (rate)**	**Major findings**
**Discharge prescriptions**			

Coombes et al. 2004 [15]	605 medications on 100 hand written prescriptions	30 (5.0% of medications)	The most common types of errors were omissions (2.6%) and dosing errors (0.8%).

Coombes et al. 2004 [15]	700 medications on 100 computer generated prescriptions	81 errors (11.6% of medications)	The most common types of errors were dosing errors (3.6%), duration errors (1.9%), medication not required on discharge (2.1%) and omissions (1.7%).

**Inpatient and discharge prescriptions from medical and surgical wards assessed**			

Coombes et al., 2001 [49]	2978 prescriptions	71 (2.4%)errors with potential to cause an ADE	The most common error types found were wrong or ambiguous dose (1.0% of prescriptions), dose absent from prescription (0.6% of prescriptions), frequency absent from prescription (0.4% of prescriptions*)

**Medication charts in a paediatric department assessed**			

Dawson et al., 1993 [50]	212 medication charts^#^	52 major errors** (24.5% of med'n charts)	The most common error types were dose errors (12.3% of charts reviewed), error of administration frequency (5.7% of charts reviewed), error of administration route (5.2% of charts reviewed), error in drug name/formulation (1.4% of charts reviewed).

Dawson et al., 1993 [50]	325 medication charts^#^	35 major errors** (10.8% of med'n charts)	The most common error types were dose errors (4.9% of charts reviewed), error of administration route (2.5% of charts reviewed), error of administration frequency (1.8% of charts reviewed), error in drug name/formulation (1.5% of charts reviewed).

**Errors in medical, surgical, children's wards and a critical care unit assessed**			

Leversha, 1991 [51]	6641 medication chart checks	241 (3.6% of chart checks)	Prescribing errors detected were incorrect dose (1.2% of chart checks), no strength specified (1.0%), insufficient information (0.2%). It was also found that failure to record the patient's current (ongoing) medication on the chart occurred in 69 cases (1.0% of chart checks)

**Prescriptions presenting to pharmacy department assessed**			

Fry et al., 1985 [52]	10 562 prescriptions	574 (5.4%),	Included assessment of legal requirements, (eg patient name and address, doctor's signature) as well as clinical requirements (eg dose, frequency,) The strength was missing or incorrect in 0.7%, the directions inappropriate or omitted in 0.4%, and the wrong drug in 0.06%.

* Percentage of prescriptions for regular and 'as required" medications only; ** Major errors included errors in drug name, dose, formulation, route or frequency of administration; ^#^Note: unit of analysis is medication chart, which may include one or more prescriptions.

One study was located that assessed documentation of medicines by emergency department doctors compared to the medication history taken by a pharmacy researcher, finding very high rates of discrepancy. Emergency department doctors documented only 16% of the medicines subsequently documented by the pharmacist researcher. This was primarily due to the fact that when the emergency department doctor had documented on the emergency department admission form "see accompanying medication list", rather than rewriting the medicines on to the form, the medication was classified as omitted [16]. While this method is not directly comparable to studies that have used chart review to compare histories taken by different health professionals, the results of this study highlight the potential for error in the emergency department due to poor documentation and potential for forms and lists to be separated. Another study, also undertaken in the emergency department, assessing medication errors prior to an intervention, found 88 errors amongst 56 patients over a five day period. On average the patients were prescribed 7.2 medicines, suggesting a very high error rate of 22% [17].

While not assessing errors, one study assessed the quality of opioid prescribing, finding that 90% of prescribing orders did not comply with at least one of 13 quality statements that had been developed to assess performance [18]. It should be noted that not all of the quality statements would necessarily be judged as inappropriate prescribing, however, the study does highlight that documentation of opioid prescribing could be improved.

Two other relevant studies included one that assessed whether patients were weighed in hospital prior to prescription of renally excreted medicines [19] and another looking at the dosage of medicines in people with renal failure [20]. Failure to weigh patients who are prescribed renally excreted medicines has been identified as a risk for medication error. The NSW study included patients admitted over a three month period to one medical ward and one surgical ward. Only 26% of the 38 persons prescribed renally excreted medicines were weighed prior to prescription. Although only small numbers, the study also reported a significant increase in bleeds amongst those prescribed anticoagulants who were not weighed compared to those who were weighed (p = 0.03) [19].

A retrospective study of 192 patients admitted to a Queensland hospital over a four month period with a creatinine clearance of 40 ml/min or less found that 45% of prescriptions for renally excreted medicines had an inappropriately high dose, with the majority of these being present on admission [20].

#### Factors contributing to prescribing errors

There have been a number of studies assessing factors contributing to prescribing error resulting in a much stronger Australian evidence base for the contribution of systems factors to medication errors.

A qualitative study undertaken in Queensland examining reasons for 21 prescribing errors by hospital interns found causation was multifactorial with a median of four (range 2–5) types of factors contributing to error [21]. Environmental factors contributed in 19 (90%) cases; team factors contributed in 16 (76%) cases; individual factors contributed in 16 (76%) cases; task factors contributed in 16 (76%) cases and patient factors contributed in 13 (62%) cases. As the study was qualitative these percentages should be considered indicative only. Environmental factors included issues such as staffing levels, skill mix, workload, workflow design, administrative and managerial support. Task factors included issues such as the medication chart design, protocols and availability and accuracy of test results. Individual factors included knowledge and skills, motivation, and individual health. Team factors included issues such as communication, supervision and structure, while patient factors included patient condition and communication ability [21].

These results were confirmed in a Western Australian study which explored 29 medication errors, with 21 of these errors being due to a slip/lapse error [22]. The 11 administration or dispensing errors were all slip/lapse errors; 10 of the prescribing errors were slip/lapse and eight were knowledge based errors. Individual, team, patient and environmental factors were all implicated in contributing to the error. The authors noted "errors were more likely to occur during tasks being carried out after hours by busy, distracted staff, often in relation to unfamiliar patients" [22]. Communication problems and difficulty accessing information were noted to contribute to prescribing errors [22].

The contribution of the delivery of information has also been assessed in a Victorian study, which found that it was not the availability of the information that was the problem but inaccessibility to on-line information and lack of connectivity between applications that caused problems [23]. In this study, electronic prescribing, ordering and dispensing systems were available as were electronic clinical and scheduling management systems and electronic systems for managing test and radiology results, again highlighting the contribution of environmental factors to error.

### Administration errors in acute care

#### Incidence of administration errors

There were no new studies located since 2002 that assessed the overall incidence of administration errors, however, one study analysed rates of omitted medicines [24] and another assessed error rates for IV administration [25] (Table 5). Other studies of administration errors that were located relate to insulin administration [26], and administration of "when required" medicines [27,28].

**Table 5:** Medication administration errors: Australian hospitals 1988–2007

	**Total opportunities for error**	**Error rate (excluding minor timing errors)**	**Type of medication error**				
			
			**Timing error**	**Wrong dose**	**Omission**	**Wrong formul'n or route**	**Other**
**WARD STOCK-BASED SYSTEMS**							

Stewart et al., 1991 [53]	2017	369 (18.3%)	75 (3.7%)	46 (2.3%)	82 (4.1%)	6 (0.3%)	160 (7.9%)

McNally et al., 1997 [54]	494	76 (15.4%)	22* (4.5%)	20 (4.0%)	13 (2.6%)	2 (0.4%)	19 (3.8%)

Lawler et al. 2004 [24]	4887	Omission only assessed			369 (7.6%)		

**COMBINATION SYSTEMS**							

Rippe and Hurley, 1988 [55]	312	52 (16.7%)	24 (7.7%)	6 (1.9%)	12 (3.8%)	3 (0.96%)	7 (2.2%)

Camac et al., 1996 [56]	370^†^	47 (12.7%)	25 (6.8%)	N/G^‡^	N/G^‡^	N/G^‡^	N/G^‡^

**INDIVIDUAL PATIENT SUPPLY**							

de Clifford et al., 1994 [57]	164	10 (6.1%)	1 (0.6%)	2 (1.2%)	5 (3.0%)	0	2 (1.2%)

McNally et al., 1997 [54]	502	24 (4.8%)	12* (2.4%)	2 (0.4%)	7 (1.4%)	0	3 (0.6%)

Thornton and Koller 1994 [58]	242	20 (8.3%)	2 (0.8%)	0	13 (5.4%)	0	5 (2.1%)

**IV FLUID ADMINISTRATIONS**							

Han et al., 2005 [25]	687	124 (18%)					

* Major timing errors included, minor timing errors excluded – a deviation of 2 or more hours from the ordered time. All other studies define a 'timing error' as a deviation of one or more hours from the ordered time.

† Total data using two different storage sites – ward bay medication drawer and patient's bedside locker.

‡ N/G – insufficient data given to calculate rate of individual error types

A small study involving 67 inpatients with a total of 4887 medication administrations found an omission of medicine rate of 7.6% (369 cases). Omission was defined as complete omission (i.e. the dose was not given before the next dose of medicine was due). Nurse initiated and when required doses were excluded. In the majority of cases, 74% (273 cases), the reason for omission was documented, with most documented as withheld (84 cases), refused (63 cases), unable to accept (51 cases) and fasting (33 cases). One hundred and twenty cases were assessed for severity on a scale from zero to 10 where zero = no harm and 10 = death. The majority of cases were scored at two or less [24].

A study made 687 observations of 639 intravenous fluid administrations in 3 surgical wards across a four week period in 2003. Observations were made between 0900 and 1600 as well as 2000 to 0300. Eighteen percent of observations were associated with a medication error. Of these, 79% of errors were incorrect administration rate. The predominant factor associated with increased error rate was the presence of a peripheral line (OR 3.5, 95%CI 1.9–6.5), while IV infusion control devices (OR 0.12, 95%CI 0.06–0.25), nasogastric feeds (OR 0.09, 95% CI 0.01–0.64) and permanent staff (OR 0.48, 95% CI 0.31–0.76) were predominant factors associated with decreased risk [25].

One observational study assessing 195 insulin administrations over two months found blood glucose testing was undertaken within 30 minutes of the insulin dose in only 22% of cases for rapid acting insulin and 41% of cases for conventional insulin, while 94% of rapid acting insulin doses were administered within an acceptable time of the meal delivery, compared to only 43% of conventional insulin doses [26]. This study excluded long acting insulins, incomplete or illegible records and all those in palliative care.

Two studies assessed "when required" medication administration orders finding that documentation was often inadequate [28]. One study assessing paracetamol orders in children found that lack of documentation resulted in miscommunication between doctors and nurses, with different understandings of the intention for use and when to use [28]. Another study assessing psychotropic medication use amongst 43 patients in a psychiatric unit found on 9% of occasions no reason for use was recorded, on 39% of occasions it could not be determined who initiated the request for medicine and on 41% of occasions no outcome of the effect was recorded [27].

#### Factors contributing to administration errors

As with prescribing errors, there are now studies assessing factors contributing to administration errors resulting in a much stronger Australian evidence base for the contribution of systems factors to medication errors.

One Victorian study surveyed 154 registered nurses employed in regional hospitals, with 79 (51%) respondents [29]. Interruptions and distractions were the most common environmental factors cited by 25% as contributing to error, followed by poor communication (13%). The most common human factor cited was stress/high workload (25%) followed by fatigue/lack of sleep (17%). Twenty nine percent of respondents agreed with the statement "I need further training in medication administration" [29]. These results were confirmed in a Queensland study also involving nurses working in rural or remote areas [30]. High workloads, low staffing levels and high doctor expectations were all associated with a higher rate of errors, while higher levels of knowledge were found to be protective against errors [30]. A further study demonstrated how individual distress impacted on violations (deviation from rules) which in turn impacted on error rates [31]. Individual distress however, was in turn affected by factors such as organizational climate and quality of work life [31], again emphasizing the importance of the system to error prevention. Information flow was found to be a problem for nurses in a qualitative study involving paediatric nurses, with difficulty using computers and physically accessing computer terminals because of their location and number identified as an issue [32]. Similarly, policy adherence was reported to be affected by the busyness of the ward, with less policy adherence when wards were busiest [32]. Another qualitative study found that nurses were more likely to assess patients prior to medication administration rather than after administration, with assessment of the effect of the medication more likely to be limited to symptomatic therapy (eg pain relief) than other therapies [33], and that this was often poorly documented [34].

## Conclusion

Approximately 2%–3% of Australian hospital admissions are medication related. This represents an estimated 190,000 medication related hospital admissions per year, with estimated costs of $660 million. Of the studies that have assessed preventability, estimates remain relatively consistent with approximately 50% potentially preventable. There are now data suggesting that adverse events associated with within hospital transfer are also high.

Results of incident reporting from hospitals show consistent results in South Australia, Western Australia and New South Wales. Medication remains the second most common type of incident reported. Omission or overdose of medication is the most frequent type of medication incident reported and analgesics and anticoagulants are the medicines most commonly implicated.

One new study since 2002 assessed the overall incidence of prescribing errors on discharge prescriptions finding an error rate of 11.6% for computer generated prescriptions compared with 5.0% for hand written prescriptions. The findings suggest that computerised prescribing systems without decision support may not reduce prescribing errors. Similarly, systems studies suggest implementation of computer systems without attention to connectivity, work flow and staff training will not resolve errors. Studies conducted on prescribing of renally excreted medications suggest that there are high rates of prescribing errors in patients requiring monitoring and medication dose adjustment. There were no new studies located that assessed overall administration or dispensing error rates in acute care.

In comparison to 2002, there is now a much stronger Australian research base demonstrating that systems factors are contributing to medication errors, with team, task, environmental, individual and patient factors contributing to error. Environmental factors include issues such as staffing levels, skill mix, workload, workflow design, administrative and managerial support. Task factors include issues such as the medication chart design, protocols and availability and accuracy of test results. Individual factors include knowledge and skills, motivation, and individual health. Team factors include issues such as communication, supervision and structure, while patient factors include condition and communication ability.

Overall, data from this review indicate that problems with medication safety in the acute care setting still represent a major challenge to the Australian health care system. As has been recognised from earlier research, there are multiple factors that contribute to medication errors and other problems with medicines within this setting. Understanding the contributing system factors that underlie medication errors can assist the development of strategies and policies that tackle these factors on a variety of levels. There is an ongoing need for strong leadership and commitment from governments, health care managers and professionals and consumers to make improved medication safety a priority in Australia. There is a need to support strategic research which continues to monitor the rates of medication problems in the Australian setting as new strategies are implemented and which will help to identify new issues as they arise. Part two of this review examines the Australian evidence base for the use of various approaches which may help to build safer systems and reduce medication problems.

## Abbreviations

ADR: (adverse drug reaction); ADE: (adverse drug event); CI: (confidence interval); CNS: (central nervous system); OR: (odds ratio); N/A: (not assessed); NSAID: (non-steroidal anti-inflammatory drug); NSW: (New South Wales); SA: (South Australia); WA: (Western Australia).

## Competing interests

The authors declare that they have no competing interests.

## Authors' contributions

EER was the main author of Part 1 of this review and was involved in reviewing the literature, summarising study findings and synthesis of the findings with those from the previous medication safety review. SJS was responsible for the drafting and editing of this paper and contributed to the review of the relevant literature.

## Authors' information

EER is an Associate Professor and co-director in the Quality Use of Medicines and Pharmacy Research Centre (QUMPRC), Sansom Institute, University of South Australia. SS is a Research Fellow in the QUMPRC. EER and SS were the primary authors of the *Second National Report on Patient Safety Report – Improving Medication Safety *for the Australian Council for Safety and Quality in Health Care in 2002.

## Supplementary Material

Additional file 1 **Additional file table S1; Medication-related hospital admissions or readmissions: Australia 1988 – 2007**. Table showing the medication-related hospital admissions or readmissions in Australia from 1988 – 2007. Click here for file
