# Supplementary material for: Medication safety in acute care in Australia: where are we now? Part 1: a review of the extent and causes of medication problems 2002–2008
Source: Aust New Zealand Health Policy. 2009 Aug 11;6:18. doi: 10.1186/1743-8462-6-18 (PMC2733897; doi:10.1186/1743-8462-6-18)
Supplement: Additional file 1 — Additional file table S1; Medication-related hospital admissions or readmissions: Australia 1988 – 2007. Table showing the medication-related hospital admissions or readmissions in Australia from 1988 – 2007. [file 1743-8462-6-18-S1.doc]

**Table S**1: Medication-related hospital admissions or readmissions: Australia 1988 - 2007

|  | | **Total admissions reviewed** | | **Total medicine related** | **Type of medicine related admission** | | | | | |
| --- | --- | --- | --- | --- | --- | --- | --- | --- | --- | --- |
|  |  |  |  |  | **Adverse drug reaction** | **Non-compliance** | | **Over-dose** | | **Other** |
| **All hospital admissions assessed** | | | | | |  |  | | |  |
| Carroll et al., 2003 [1] | | 50712 | | 643 (1.27%) | 643 (1.27%) | N/A | | N/A | | N/A |
| Gleeson 1988 [2] | | 947 | | 34 (3.6%) | 34 (3.6%) | N/A | | N/A | | N/A |
| Larmour et al 1991 [3] | | 5623 | | 136 (2.4%) | 90 (1.6%) | 5 (0.09%) | | 40 (0.7%) | | 1 (0.02%) |
| **Admissions via Emergency Department assessed** | | | | | |  |  | | |  |
| Galbraith 1993 [4] | | 751 | | 48 (6.4%) | Unknown | Unknown | | 7 (0.9%) | | Unknown |
| Dartnell et al 1996 [5] | | 965 | | 68 (7%) | 26 (2.7%) | 15 (1.6%) | | 13 (1.3%) | | 14 (1.5%) |
| **Admissions to Medical Wards assessed** | | | | | |  |  | | |  |
| Sarkawi & Daud 1995 [6] | | 419 | | 49 (11.7%) | 21 (5%) | 12 (2.9%) | | 14 (3.3%) | | 2 (0.5%) |
| Stanton et al.1994 [7] | | 691 | | 81 (11.7%) | 21* (3%) | 10* (1.4%) | | 26* (3.8%) | | 11* (1.6%) |
| Leishman & Vial 1998^a^ [8] | | 217 | | 33 (15.2%) | 10 (4.6%) | 8 (3.7%) | | 11 (5.1%) | | 4 (1.8%) |
| **Unplanned readmissions assessed** | | | | | |  |  | | |  |
| Blackbourn 1991 [9] | | 180 | 29 (16%) | | 12 (6.7%) | 14 (7.8%) | | 1 (0.6%) | | 2 (1.1%) |
| Hewitt 1995 [10] | | 131 | 46 (35%) | | 29 (22%) | 1 (0.8%) | | 0 | | 16 (12.2%) |
| Greenshields et al., 1997 [11] | | 63 | 17 (27%) | | unknown | unknown | | unknown | | unknown |
| Stowasser et al., 2000^a^ [12] | | 28 | 9 (32.1%) | | unknown | unknown | | unknown | | unknown |
| **Paediatric admissions assessed – medical only excluding oncology** | | | | | | | | | |  |
| Easton, 1998 [13] | | 1682 | 58 (3.4%) | | 10 (0.6%) | 29 (1.7%) | | 10 (0.6%) | | 9 (0.5%) |
| Easton et al 2004 [14] | | 2933 | 127 (4.3%) | | 29 (1.0%) | 38 (1.3%) | |  | |  |
| **Geriatric admissions via emergency departments assessed** | | | | | | | | | |  |
| Ng 1996 [15] | | 172 | 31 (18%) | | 18 (10.5%) | 5 (2.9%) | | 1 (0.6%) | | 7 (4.1%) |
| Atkin et al 1994 [16] | | 217 | 48 (22.1%) | | 41 (18.9%) | 5 (2.3%) | | 1 (0.5%) | | 1 (0.5%) |
| Wong et al. 1993 [17] | | 245 | 49 (20%) | | 35 (14.3%) | 13 (5.3%) | | 1 (0.4%) | | N/A |
| Wong et al. 1993 [17] | | 541 | 81 (15%) | | 61 (11.3%) | 19 (3.5%) | | 1 (0.2%) | | N/A |
| Harding, 1998 [18] | | 16 | 6 (37.5%) | | 4 (25.0%) | 1 (6.25%) | | 0 | | 1 (6.25% |
| Chan et al., 2001 [19] (>=75 years) | | 240 | 73 (30.4%) | | 32 (13.3%) | 9 (3.8%) | | 1 (0.42%) | | 31 (12.9%) |
| **Cardiac patients admitted to the coronary care unit or medical wards** | | | | | | | | | | |
| Lee & Oldenburg 1993 [20] | 112 | | 37 (33%) | | 14 (12.5%) | 11 (9.8%) | | 0 | 12 (10.7%) | |
| **All admissions: ADRs during hospital stay or on admission** | | | | | | | | | | |
| Carroll et al., 2003 [1] | | 50712 | |  | 1389 2.7% | N/A | | N/A | | N/A |
| **Oncology patients ADRs during hospital stay or on admission** | | | | | | | | | | |
| Lau et al., 2004 [21] | 171 | | 127 (74.3%) | | 127 (74.3%) | N/A | | N/A | N/A | |
| **Emergency department attendances** | | | | | | | | | | |
| Galbraith 1993 [4](adults) | 594 (not admitted) | | 51 (8.6%) | | 8 (1.3%) |  | |  |  | |
| Easton 2003 [22] (paediatrics) | 8601 (includes admissions) | | 280 (3.2%) | | 118 (1.4%) |  | |  |  | |
| Hendrie et al., 2007 [23] | 3332 (incl. admissions) | | 45 (1.4%) | | 45 (1.4%) |  | |  |  | |

N/A = Not assessed; ADRS = adverse drug reactions

* only definite or probable drug-related admissions reported (all other results report definite, probable or possible drug related admissions); ^1^  medical and respiratory wards and endocrinology unit; ^a^ assessed by medical file review and examination of medication changes
